# Current global status, subtype distribution and zoonotic significance of *Blastocystis* in dogs and cats: a systematic review and meta-analysis

**DOI:** 10.1186/s13071-022-05351-2

**Published:** 2022-06-22

**Authors:** Morteza Shams, Laya Shamsi, Amirhosein Yousefi, Alireza Sadrebazzaz, Ali Asghari, Behnam Mohammadi-Ghalehbin, Saeed Shahabi, Gholamreza Hatam

**Affiliations:** 1 Zoonotic Diseases Research Center, Ilam University of Medical Sciences, Ilam, Iran; 2 Department of Pathobiology, Faculty of Veterinary Medicine, Urmia University, Urmia, Iran; 3 Department of Medical Lab Science, School of Medicine, Ardabil Branch, Islamic Azad University, Ardabil, Iran; 4 Education and Extension Organization, Razi Vaccine & Serum Research Institute, Agricultural Research, Mashhad, Iran; 5 Department of Parasitology and Mycology, School of Medicine, Shiraz University of Medical Sciences, Shiraz, Iran; 6 Zoonoses Research Center (ZRC), Ardabil University of Medical Sciences, Ardabil, Iran; 7 Department of Biology and Control of Disease Vectors, School of Health, Shiraz University of Medical Sciences, Shiraz, Iran; 8 Basic Sciences in Infectious Diseases Research Center, Shiraz University of Medical Sciences, Shiraz, Iran

**Keywords:** *Blastocystis*, Prevalence, Subtypes, Distribution, Dogs, Cats, Systematic review, Meta-analysis

## Abstract

**Background:**

*Blastocystis* is a common intestinal protozoa found in animal and human fecal samples, with over 1 billion individuals infected worldwide. Since domestication, dogs and cats have had a close bond with humans. However, their close proximity poses a potential health risk since they may harbor several zoonotic agents. A global estimate of *Blastocystis* infection and subtype (ST) distribution in dogs and cats would therefore be of great health importance to humans.

**Methods:**

We performed a comprehensive systematic search of four English-language databases (PubMed, Scopus, Google Scholar, Web of Science) for relevant articles up to 8 November 2021. The random-effects model was used to make pooled estimates with confidence intervals (CIs).

**Results:**

In total, we identified 49 publications that met our inclusion criteria and subsequently analyzed the 65 datasets in these articles, of which 23 and 42 datasets were on cats and dogs, respectively. Among the 2934 cats included in the 23 datasets, which involved 16 countries, the prevalence rate of *Blastocystis* infection was 9.3% (95% CI 5.3–15.9%). The prevalence of *Blastocystis* infection was slightly lower [7%, 95% CI 4.7–10.4%) among the 7946 dogs included in the 42 datasets, involving 23 countries. The sensitivity analysis showed that no remarkable variation in the estimates upon the stepwise removal of each dataset. Higher ST diversity was found among the examined dogs (ST1-8, ST10, ST23, ST24) than among cats (ST1-4, ST10, ST14). Among dogs, ST3 was the most frequent ST (41.3%), followed by ST2 (39.3%), ST1 (30.9%), ST4 (13.4%), ST8 (12.7%), ST10 (11%) and ST5 (8.1%). Also among dogs, each of ST6, ST7, ST23 and ST24 was observed in only one study. Of the ST found in the cats examined, ST4 (29.5%), followed by ST10 (22.5%), ST1 (19.8%) and ST3 (17.6%) were the most common. A single study also reported the presence of both ST2 and ST14 in cats. With respect to zoonotic *Blastocystis* STs (ST1–ST9 and ST12), eight were reported from dogs (ST1-ST8) and four were isolated from cats (ST1–ST4), showing the implication of dog and cats in zoonotic transmission.

**Conclusions:**

Taken together, our results show that elucidation of the true epidemiology and ST distribution of *Blastocystis* in dogs and cats demands more comprehensive studies, particularly in the negelected regions of the world.

**Graphical Abstract:**

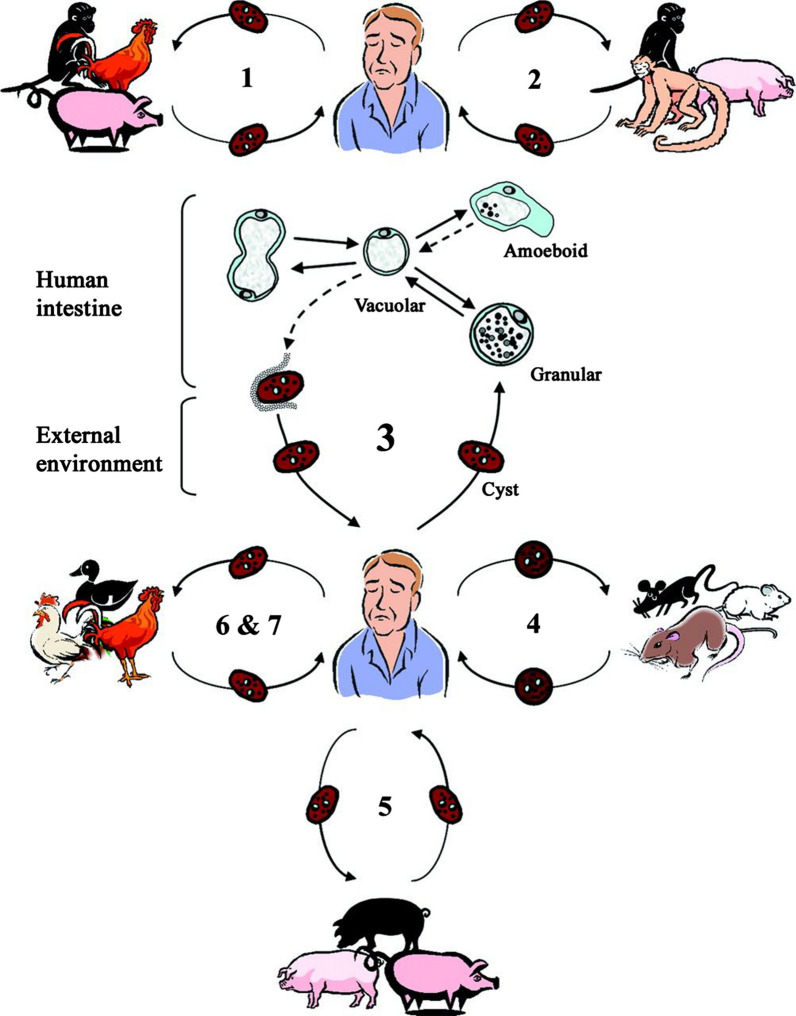

**Supplementary Information:**

The online version contains supplementary material available at 10.1186/s13071-022-05351-2.

## Background

*Blastocystis* is a common enteric protozoa found in fecal samples of humans and animals. Over 1 billion people are infected globally [1, 2]. Four major life stages have been described in this polymorphic parasite, comprising vacuolar, granular, amoeboid and cyst stages; among these, the avacuolar and multivacuolar forms are less common during encystation or excystation [3, 4]. General consensus on the transmission of *Blastocystis* and is that infection occurs through the fecal–oral route with the ingestion of cyst-contaminated water or food [5]. Zoonotic transmission may also be possible through close animal-human contact, but the extent and frequency of such events remain largely unknown, requiring more in-depth investigation [6, 7]. Symptomatic human infections may manifest as diarrhea, abdominal pain, flatulence, inflammatory bowel disease, irritable bowel syndrome (IBS) and cutaneous lesions (urticaria) [8, 9]. Nevertheless, it is not fully known whether *Blastocystis* possesses pathogenic potential since carriage state is highly frequent [10].

Microscopy, culturing and molecular assays are the primarily methods used to detect *Blastocystis* infection in hosts. However, the discrimination of subtypes (STs) is only possible using DNA-based methods and sequence analysis of the small subunit ribosomal RNA (SSU rRNA) gene [2, 11]. A total of 32 phylogenetically distinct *Blastocystis* subtypes have been proposed based on SSU rRNA analysis, including zoonotic STs (ST1–9, ST12) and STs isolated only from animals (ST10, ST11, ST13–17, ST21, ST23–32). Some experts have noted that ST18-20 and ST22 are invalid due to ambiguities in the 5′ and 3′ ends of the SSU rRNA sequences. Nevertheless, according to the criteria currently in place to qualify as a unique subtype, a total of 28 subtypes (ST1–17, ST21 and ST23–32) are generally widely recognized as being valid subtypes [12–14].

The One-Health concept is an integrated approach to human healthcare that considers human health to be closely connected to animal health and the environment, proposing that each constituent (e.g. animals) may play a principal role in transmission dynamics of *Blastocystis* [15]. Dogs and cats, as important pet animals, may harbor zoonotic agents and be considered potential reservoirs for *Blastocystis*. Hence, detection of *Blastocystis* infection in these animals is important for improving human health levels. We performed the present systematic review and meta-analysis to clarify the global epidemiology, subtype distribution and zoonotic importance of this parasitic protozoan in dogs and cats.

## Methods

### Study design and reporting protocol

A systematic review and meta-analysis of the worldwide epidemiology, subtype distribution and zoonotic importance of *Blastocystis* was designed and implemented in 2021, with dogs and cats as the target population. The reporting protocol was designed based on the Preferred Reporting Items for Systematic Reviews and Meta-Analysis (PRISMA) guideline [16].

### Databases and search strategies

A comprehensive search of four electronic databases (PubMed, Scopus, Google Scholar, and Web of Science) was conducted by two of the authors (AA and MSH) for relevant articles published up to 8 November 2021, using the keywords: “*Blastocystis*,” “*Blastocystis* sp.,” “Subtypes,” “Prevalence,” “Epidemiology,” “Frequency,” “Occurrence,” “Dog,” “Cat,” “Canine” and “Feline,” with “OR” and/or “AND” operators. To expand the search for relevant publications, additional keywords were also used and the reference lists of identified papers were explored. The titles and abstracts of the identified publications were reviewed, duplicated papers were removed and the full-text of each article identified as being relevant was obtained. The eligibility of the papers was evaluated independently by six of the authors (GH, BM, LSH, AY, AS, SSH); any disagreement was resolved through consultation with the leading reviewer (AA).

### Eligibility criteria

Observational cross-sectional studies reporting the prevalence and/or subtypes of *Blastocystis* in dogs and cats utilizing microscopy of stool samples and/or molecular techniques up to 8 November 2021 were included in present systematic review. Excluded from this systematic review and meta-analysis were case reports, reviews, letters, studies on humans or other animals, studies involving experimentally infected animals, studies without *Blastocystis* prevalence rates and studies containing unclear/confusing information.

### Quality assessment and data extraction

The Joanna Briggs Institute (JBI) critical appraisal checklist for studies reporting prevalence data was used for qualitative evaluation of the articles [17]. Articles were included in this systematic review and meta-analysis if they were assessed to have checklist scores of 4–6 points (moderate quality) or 7–9 points (high quality); papers with a checklist score of ≤ 3 points were excluded. The following items were extracted using a pre-piloted checklist for each study: the first author’s last name, quality assessment score, publication year, implementation year, country, continents, WHO regions, related STs, total sample size and infected sample size. In the current review, information regarding WHO regions was obtained from the relevant WHO URL (https://www.who.int/standards/classifications).

### Data analysis

The extracted data were exported to the Comprehensive Meta-Analysis (CMA) version 3 software for meta-analysis, with *P* < 0.05 considered to be a statistically significant value [18]. A Forest plot diagram was designed using a random-effects model to represent the weighted frequencies with 95% confidence intervals (CIs). The I^*2*^ index was used to assess heterogeneity between included studies, ranging from < 25% (low variation) and 25–50% (moderate variation), to > 50% (high variation) [19]. The subgroup analysis of the pooled prevalence of the parasitic infection among dogs and cats was performed based on publication year, WHO region, country, continent and sample size. Additionally, variations in the final weighted prevalence of *Blastocystis* infection upon stepwise removal individual studies were assessed by sensitivity analysis. Meta-regression was performed to evaluate the likely association between some variables (publication year and sample size) and *Blastocystis* frequency among examined animals. The funnel plot was used to check the probability of publication bias during the analysis.

## Results

### Description of the systematic search and article selection

**Fig. 1:**
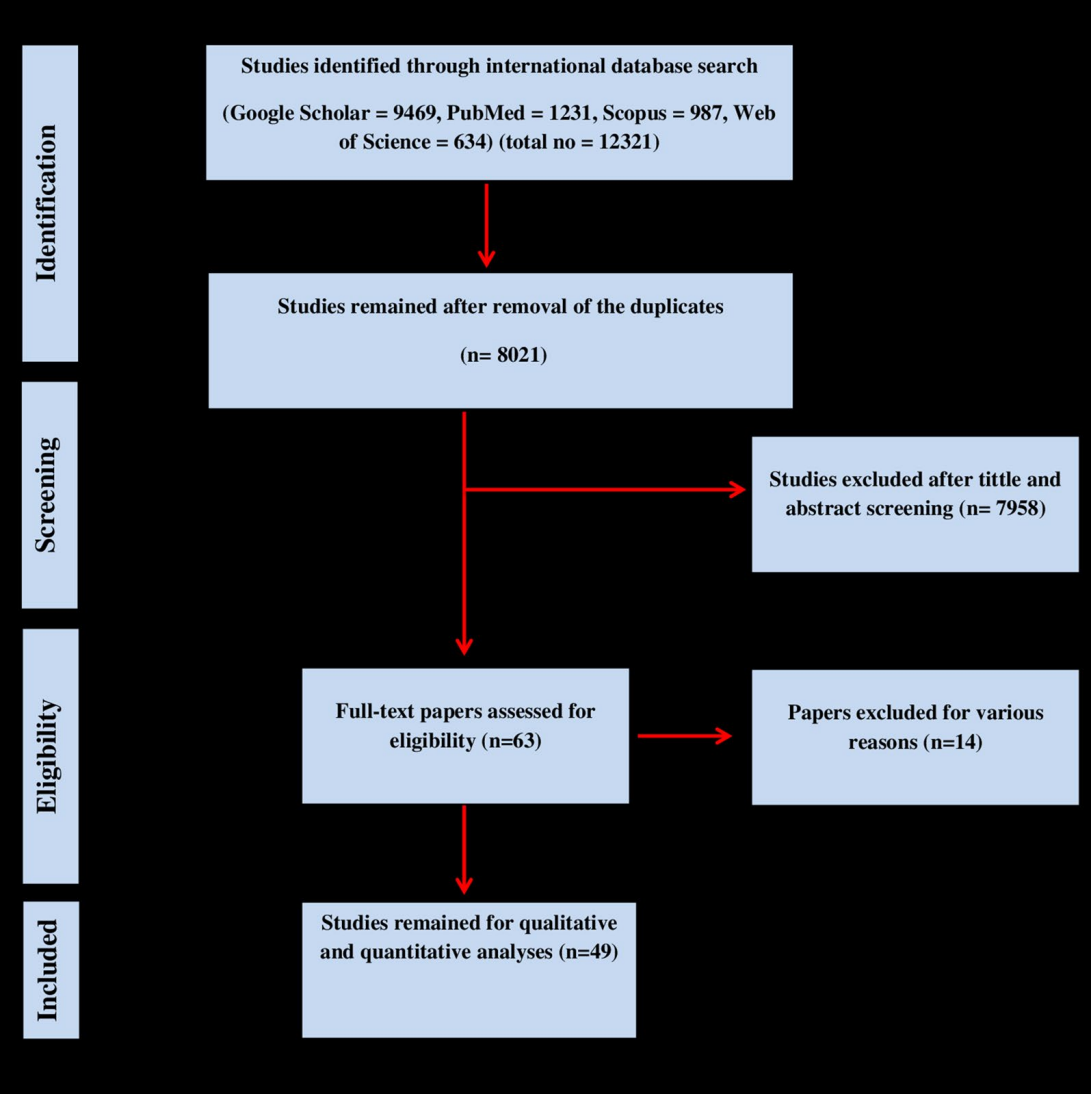
Flowchart of the included eligible studies in the present study

The strategy for the systematic search and study selection is shown in Fig. 1. In brief, 12,321 articles were identified during the primary systematic search; of these 4300 were duplicate papers and discarded, leaving 8021 articles for review of the title and abstract. Of these 8021 articles, 63 met the inclusion criteria and were fully reviewed. Qualitative evaluation using the JBI checklist resulted in the exclusion of an additional 14 articles. Ultimately, 49 studies (65 datasets) [13, 20–67] were assessed as eligible to be included in the meta-analysis (Table 1). Reasons for removing studies from the meta-analysis included animals other than dogs and cats (4 papers), intestinal parasites other than *Blastocystis* (7 articles), repetitive results (1 study) and ambiguous findings (2 papers).

**Table 1:** The main characteristics of 49 studies/papers (65 datasets) included in the present study

First author, year	Study period	Country	Total samples (*n*)	Infected samples (*n*)	Prevalence (%)	Diagnostic method	Reference
*Dogs*							
Abe, 2002	1999	Japan	54	0	0	Mic	[20]
Boutellis, 2021	2018	Algeria	9	1	11.1	Mol	[28]
Roberts, 2013	UC	Australia	56	0	0	Mol	[59]
Osman, 2015	2012–2013	France	116	4	3.4	Mol	[52]
Duda, 1998a	UC	Australia	72	51	70.8	Mic	[31]
La Sala, 2015	2012–2013	Argentina	475	14	2.9	Mic	[41]
Udonsom, 2018	UC	Thailand	13	1	7.7	Mol	[64]
Sardarian, 2015	2012	Iran	1500	1	0.1	Mic	[62]
Ramirez, 2014	UC	Colombia	40	15	37.5	Mol	[58]
Sanchez-Thevenet, 2019	2014–2016	Spain	263	3	1.1	Mic	[61]
Wang, 2013	2010–2011	Australia	80	2	2.5	Mol	[66]
Wang, 2013	2010–2011	Cambodia	80	1	1.3	Mol	[66]
Wang, 2013	2010–2011	India	80	19	24	Mol	[66]
Puebla, 2015	2014–2015	Cuba	97	2	2.1	Mic	[57]
Hurtado, 2019	UC	Colombia	421	62	14.7	Mic	[37]
Bandaranayaka, 2019	UC	Sri Lanka	50	2	1	Mic	[26]
Spanakos, 2011	2008	Greece	72	0	0	Mol	[63]
Belleza, 2016	2011–2012	Philippines	145	20	13.8	Mol	[27]
Li, 2016	2013	China	315	6	1.9	Mol	[43]
Mohaghegh, 2018	2014–2015	Iran	301	59	19.6	Mic	[47]
Ruaux, 2014	2012	USA	103	10	9.7	Mol	[60]
Higuera, 2021	UC	Colombia	4	2	50	Mol	[13]
Gazzonis, 2019	2015–206	Italy	99	21	21.2	Mol	[32]
Konig, 1997	UC	Germany	20	0	0	Culture and Sero	[39]
Leelayoova, 2009	2006	Thailand	189	5	2.6	Mic and Mol	[42]
Dalimiasl, 2001	UC	Iran	305	1	0.3	Mic	[30]
López, 2006	1996–2003	Chile	972	351	36.1	Mic	[46]
Onder, 2021	2020–2021	Turkey	200	0	0	Mol	[51]
Parkar, 2007	UC	Australia	20	2	10	Mol	[54]
Parkar, 2007	UC	Thailand	3	3	100	Mol	[54]
Awadallah, 2015	2013	Egypt	130	4	3.1	Mic	[24]
Gonzalez, 2015	2011–2012	Colombia	175	32	18.3	Mic	[34]
Gillespie, 2017	2014–2015	Australia	300	10	3	Mic	[33]
Hemalatha, 2014	2012	Malaysia	32	0	0	Mic	[35]
Noradilah, 2017	2014–2015	Malaysia	40	21	52	Mol	[49]
Liao, 2020	2018	China	651	35	5.4	Mol	[45]
Mohammadpour, 2020b	2016–2018	Iran	154	29	18.8	Mol	[48]
Paulos, 2018	2014	Spain	55	0	0	Mol	[55]
Perera, 2013	2010–2011	Sri Lanka	90	11	12.2	Mic	[56]
Mokhtar, 2018	2015–2016	Egypt	21	0	0	Mol	[22]
Wang, 2018	2015–2017	China	136	4	2.9	Mol	[67]
Villamizar, 2019	UC	Colombia	8	1	12.5	Mol	[65]
*Cats*							
Boutellis, 2021	2018	Algeria	19	12	63.1	Mol	[28]
Roberts, 2013	UC	Australia	43	0	0	Mol	[59]
Duda, 1998	UC	Australia	52	35	67.3	Mic	[31]
Udonsom, 2018	UC	Thailand	11	0	0	Mol	[64]
Pagati, 2018	UC	Indonesia	90	48	53.3	Mic	[53]
Can, 2021	UC	Turkey	465	17	3.6	Mol	[29]
Badparva, 2020	2017	Iran	120	0	0	Mol	[25]
Arbabi, 2009	2004–2005	Iran	113	19	16.8	Mic	[23]
Li, 2019	2015–2018	China	346	2	0.6	Mol	[44]
Ruaux, 2014	2012	USA	105	12	11.7	Mol	[60]
Khademvatan, 2014	2012	Iran	140	20	14.3	Mic	[38]
Konig, 1997	UC	Germany	13	0	0	Culture and Sero	[39]
Albakri, 2016	2014	Iraq	50	18	36	Mic	[21]
López, 2006	1996–2003	Chile	230	86	37.4	Mic	[46]
Okoye, 2014	2011–2012	Nigeria	119	2	1.7	Mic	[50]
Onder, 2021	2020–2021	Turkey	200	0	0	Mol	[51]
Parkar, 2007	UC	Australia	10	0	0	Mol	[54]
Kwak, 2020	UC	South Korea	158	1	0.6	Mol	[40]
Hemalatha, 2014	2012	Malaysia	24	0	0	Mic	[35]
Mohammadpour, 2020	2016–2018	Iran	119	21	17.7	Mol	[48]
Paulos, 2018	2014	Spain	34	0	0	Mol	[55]
Karakavuk, 2021	2017	Turkey	465	49	10.5	Mic	[37]
Mokhtar, 2018	2015–2016	Egypt	8	0	0	Mol	[22]

*Mic* Microscopic detection method,* Mol* molecular detection method,* Sero *serological detection method,* UC* unclear

### The quality assessment output

All of the included studies were critically appraised using the JBI quality assessment checklist adapted for cross-sectional studies. Based on the JBI score, 15 studies were of high quality (≥ 7 points) and the remaining 34 studies were of moderate quality (4–6 points) (Additional file 1: Table S1).

### Global epidemiology of *Blastocystis* infection in dogs

**Fig. 2:**
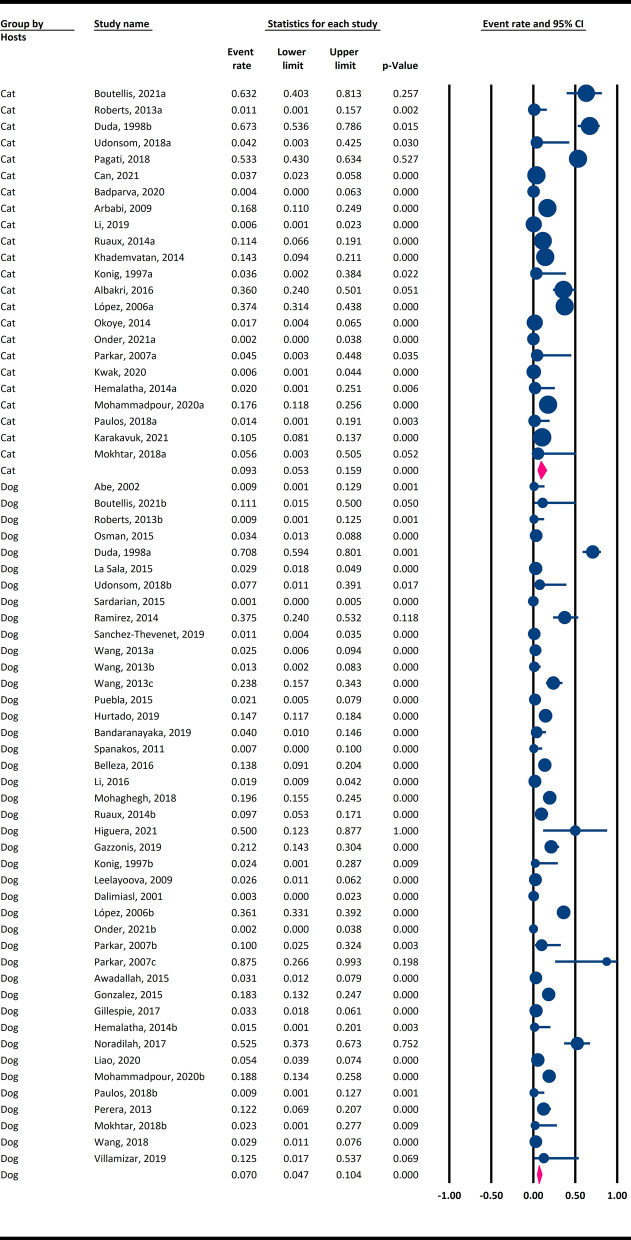
Global prevalence of *Blastocystis* spp. infection in dogs and cats using a random-effects model and 95% confidence intervals. Abbreviations: CI Confidence interval

**Fig. 3:**
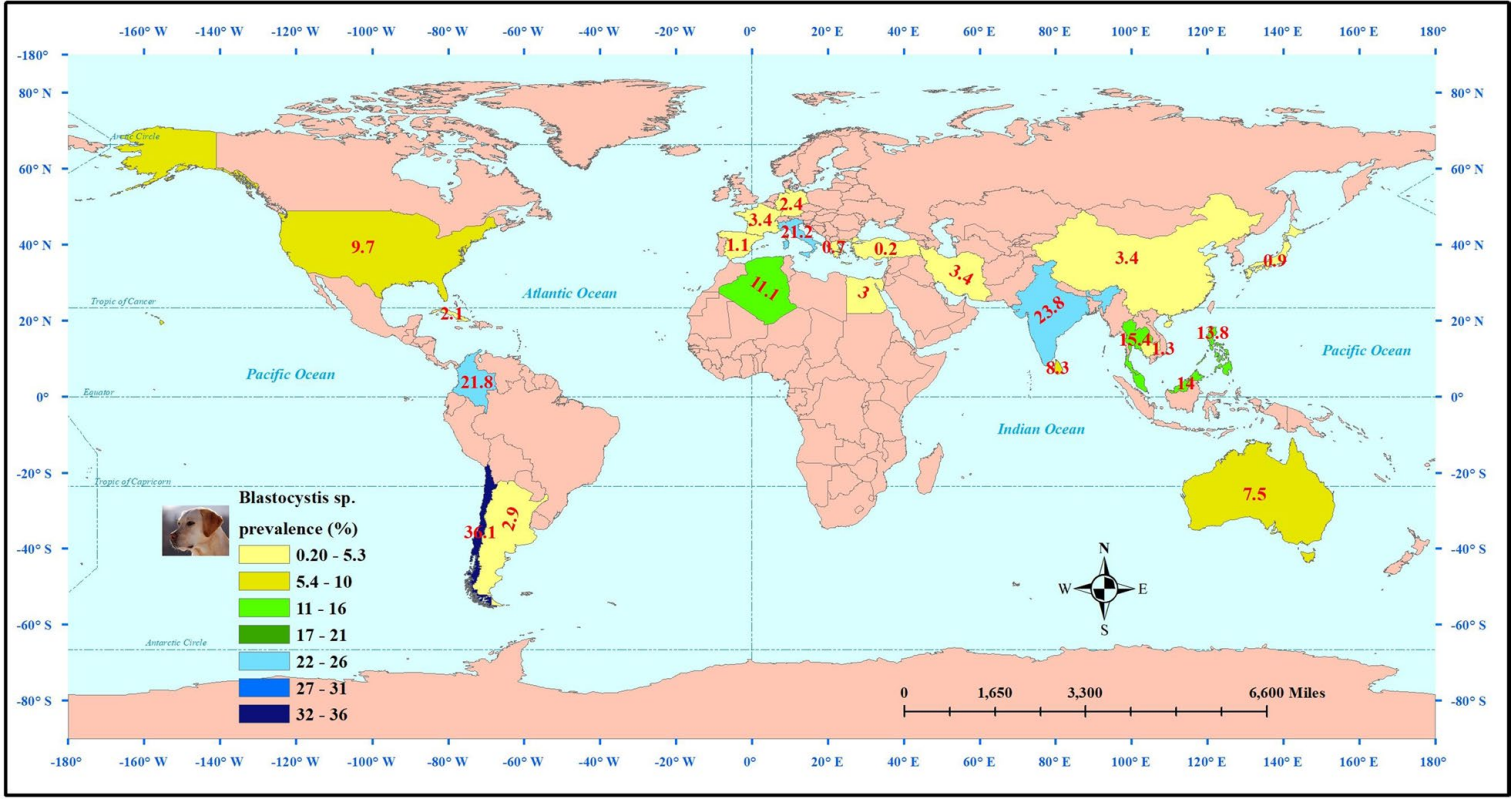
Global prevalence of *Blastocystis* spp. in dogs by country

The estimated pooled prevalence of *Blastocystis* derived from the 42 datasets on 7946 examined dogs was 7% (95% CI 4.7–10.4%) (Fig. 2). A significantly high heterogeneity was also identified among assessed studies (Cochran’s Q = 730.2, I^*2*^ = 94.4%, *P* ≤ 0.001). The global prevalence of *Blastocystis* in dogs by country is shown in Fig. 3.

### Worldwide prevalence of *Blastocystis* infection in cats

**Fig. 4:**
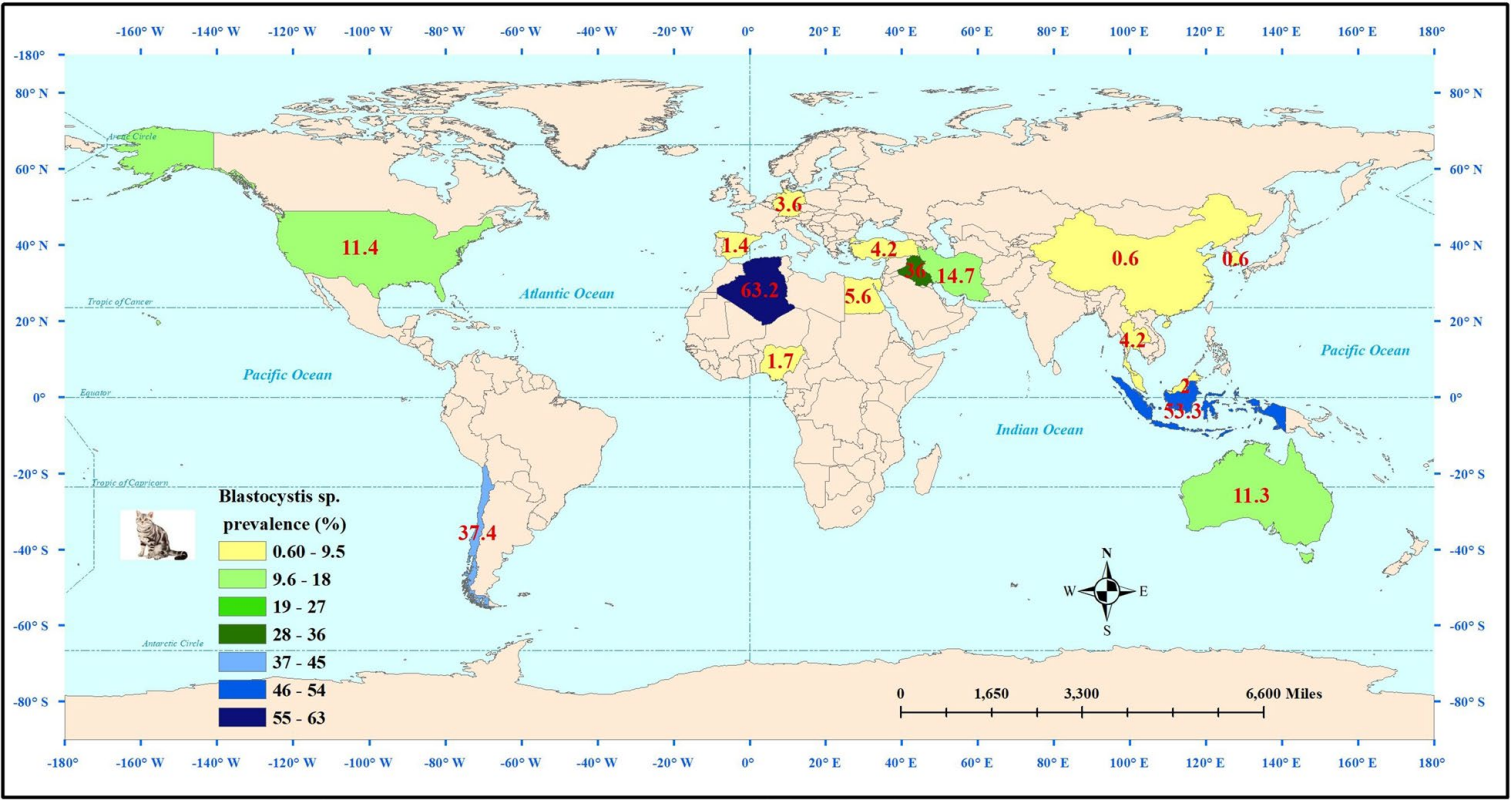
Worldwide prevalence of *Blastocystis* spp. in cats by country

The estimated weighted frequency of *Blastocystis* obtained from the 23 datasets on 2934 examined cats was 9.3% (95% CI 5.3–15.9%) (Fig. 2). A substantially high heterogeneity was reported among the assessed studies (Cochran’s Q = 350.4, I^*2*^ = 93.7%, *P* ≤ 0.001). The worldwide frequency of *Blastocystis* in cats by country is shown in Fig. 4.

### Sensitivity analysis

The sensitivity analysis showed that the stepwise removal of individual studies (i.e., each dataset) did not result in any significant variation in the final calculated prevalence (Additional file 2: Figure S1; Additional file 3: Figure S2). However, considering the omission of the studies, the prevalence of *Blastocystis* infection in dogs and cats was estimated to be between 6.3–7.7% and 8.1–10.7%, respectively.

### Overall prevalence of *Blastocystis* in dogs and cats based on investigated subgroups

The results of the subgroup analyses are shown in Table 2 and Additional files 4, 5, 6, 7, 8, 9, 10, 11, 12, 13: Figures S3, S4, S5, S6, S7, S8, S9, S10, S11, S12.

**Table 2:** Prevalence of *Blastocystis* sp. in dogs and cats based on examined subgroups

Subgroup variable	Prevalence,% (95% CI)		Heterogeneity (Cochran’s Q)		*df* (Cochran’s Q)		I^2^ (%)		*P*-value	
	Dogs	Cats	Dogs	Cats	Dogs	Cats	Dogs	Cats	Dogs	Cats
*Publication year*										
Prior to and including 2000	23.2 (0.3–96.4)	26.1 (0.7–94.6)	10	7.5	1	1	90	86.6	*P* = 0.002	*P* = 0.006
2001–2005	0.5 (0.1–2.2)	*–*	0.3	*–*	1	*–*	*–*	*–*	*P* = 0.555	*–*
2006–2010	20.1 (3.4–64.6)	22.3 (9.2–44.8)	51.2	16.8	3	2	94.1	88.1	*P* < 0.001	*P* < 0.001
2011–2015	4.5 (2.3–8.8)	6.4 (2.7–14.2)	138.1	13.6	14	4	89.8	70.6	*P* < 0.001	*P* = 0.009
2016–2021	8.4 (5.2–13.1)	7 (2.9–15.8)	189.3	217.1	18	12	90.5	94.5	*P* < 0.001	*P* < 0.001
*Continent*										
Africa	3.7 (1.6–8.4)	11.4 (0.4–80)	1.5	30.6	2	2	*–*	93.4	*P* = 0.472	*P* < 0.001
Asia	6 (3.3–10.4)	7.2 (3.5–14.2)	441.4	193.9	20	12	95.4	93.8	*P* < 0.001	*P* < 0.001
Europe	3.6 (0.8–15.5)	2.3 (0.3–14.4)	23.8	0.2	4	1	93.2	0	*P* < 0.001	*P* = 0.643
North America	5.1 (1.1–20.7)	11.4 (6.6–19.1)	4.3	0	1	0	76.6	0	*P* = 0.032	N.A
Oceania	7.5 (0.7–48.3)	11.3 (0.3–82.9)	129.9	18.5	4	2	96.9	89.2	*P* < 0.001	*P* < 0.001
South America	16 (7.7–30.4)	37.4 (31.4–43.8)	60.3	0	5	0	91.7	0	*P* < 0.001	N.A
*WHO region*										
AFR	11.1 (1.5–50)	15 (0.2–94.2)	*–*	28.9	0	1	0	96.5	N.A	*P* < 0.001
AMR	14.6 (7.5–26.3)	22.2 (6–56.1)	196.3	20.8	8	1	95.9	95.2	*P* < 0.001	*P* < 0.001
EMR	3.2 (1–9.3)	16.8 (10.1–26.6)	66.7	20.2	5	5	92.5	75.3	*P* < 0.001	*P* = 0.001
EUR	2.1 (0.5–8.8)	3.8 (1.4–9.9)	48.3	23.3	6	4	87.6	82.8	*P* < 0.001	*P* < 0.001
SEAR	11.3 (4.3–26.5)	23.4 (1.3–87.6)	31.9	5	5	1	84.3	80	*P* < 0.001	*P* = 0.025
WPR	6 (2.2–15.1)	3.1 (0.2–38.6)	262.9	95.8	12	5	95.4	94.8	*P* < 0.001	*P* < 0.001
* Country*										
Algeria	11.1 (1.5–50)	63.2 (40.3–81.3)	0	0	0	0	0	0	N.A	N.A
Argentina	2.9 (1.8–4.9)	*–*	0	*–*	0	*–*	0	*–*	N.A	*–*
Australia	7.5 (0.7–48.3)	11.3 (0.3–82.9)	129.9	18.5	4	2	96.9	89.2	*P* < 0.001	*P* < 0.001
Cambodia	1.3 (0.2–8.3)	*–*	0	*–*	0	*–*	0	*–*	N.A	*–*
Chile	36.1 (33.1–39.2)	37.4 (31.4–43.8)	0	0	0	0	0	0	N.A	N.A
China	3.4 (1.7–6.7)	0.6 (0.1–2.3)	6.5	0	2	0	69.4	0	*P* = 0.038	N.A
Colombia	21.8 (13.9–32.6)	*–*	14.9	*–*	4	*–*	73.2	*–*	*P* = 0.005	*–*
Cuba	2.1 (0.5–7.9)	*–*	0	*–*	0	*–*	0	*–*	N.A	*–*
Egypt	3 (1.2–7.3)	5.6 (0.3–50.5)	0.04	0	1	0	0	0	*P* = 0.838	N.A
France	3.4 (1.3–8.8)	*–*	0	*–*	0	*–*	0	*–*	N.A	*–*
Germany	2.4 (0.1–28.7)	3.6 (0.2–38.4)	0	0	0	0	0	0	N.A	N.A
Greece	0.7 (0–10)	*–*	0	*–*	0	*–*	0	*–*	N.A	*–*
India	23.8 (15.7–34.3)	*–*	0	*–*	0	*–*	0	*–*	N.A	*–*
Indonesia	–	53.3 (43–63.4)	*–*	0	*–*	0	*–*	0	*–*	N.A
Iraq	–	36 (24–50.1)	*–*	0	*–*	0	*–*	0	*–*	N.A
Iran	3.4 (0.9–11.7)	14.7 (9.4–22.1)	51.5	7.8	3	3	94.1	61.8	*P* < 0.001	*P* = 0.049
Italy	21.2 (14.3–30.4)	–	*–*	*–*	0	*–*	0	*–*	N.A	*–*
Japan	0.9 (0.1–12.9)	–	*–*	*–*	0	*–*	0	*–*	N.A	*–*
Malaysia	14 (0.3–91.3)	2 (0.1–25.1)	8.5	0	1	0	88.3	0	*P* = 0.003	N.A
Nigeria	–	1.7 (0.4–6.5)	*–*	0	*–*	0	*–*	0	*–*	N.A
Philippines	13.8 (9.1–20.4)	–	0	*–*	0	*–*	0	*–*	N.A	*–*
South Korea	–	0.6 (0.1–4.4)	*–*	0	*–*	0	*–*	0	*–*	N.A
Spain	1.1 (0.4–3.1)	1.4 (0.1–19.1)	0.026	0	1	0	0	0	*P* = 0.872	N.A
Sri Lanka	8.3 (2.8–21.9)	–	2.3	*–*	1	*–*	57.1	*–*	*P* = 0.127	*–*
Thailand	15.4 (1.2–73.7)	4.2 (0.3–42.5)	12.7	0	2	0	84.3	0	*P* = 0.002	N.A
Turkey	0.2 (0–3.8)	4.2 (1.3–12.6)	0	21.5	0	2	0	90.7	N.A	*P* < 0.001
USA	9.7 (5.3–17.1)	11.4 (6.6–19.1)	0	0	0	0	0	0	N.A	N.A
*Sample size*,* n*										
≤ 50	18.6 (8.6–35.8)	14.3 (5.9–30.8)	35.2	45.5	10	9	71.6	80.2	*P* < 0.001	*P* < 0.001
51–100	5.8 (2.2–14.4)	67.3 (53.6–78.6)	139.2	0	11	0	92.1	0	*P* < 0.001	N.A
101–200	6.7 (3.7–11.7)	6.7 (3.5–12.6)	58.6	38	8	7	86.3	81.6	*P* < 0.001	*P* < 0.001
201–300	2.2 (0.8–6)	37.4 (31.4–43.8)	2.7	0	1	0	63.2	0	N.A	N.A
301–400	2.8 (0.3–22.9)	0.6 (0.1–2.3)	49.4	507.8	2	0	95.9	0	*P* < 0.001	N.A
> 400	5.2 (1.5–16.4)	6.4 (2.2–17.1)	305	15.3	4	1	98.7	93.5	*P* < 0.001	*P* < 0.001

*N.A* Non-applicable

### Prevalence of each *Blastocystis* subtype in dogs

**Fig. 5:**
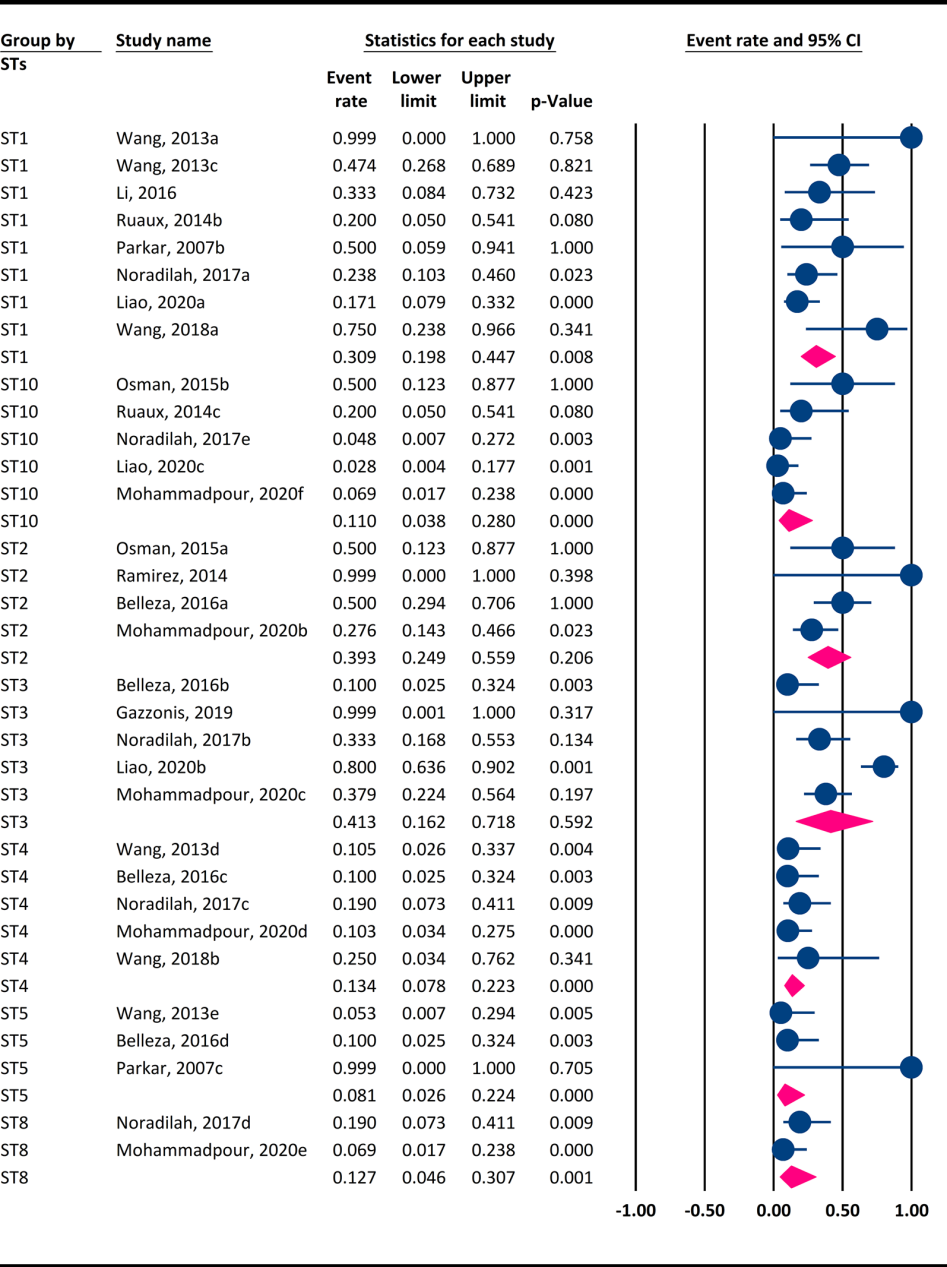
Weighted frequency of each *Blastocystis* STs in dogs using the random-effects model. Abbreviation: ST, Subtype

Among the 11 genetically diverse STs identified in dogs (ST1–8, ST10, ST23, ST24), ST3 (5 datasets; 41.3%, 95% CI 16.2–71.8%) showed the highest frequency, followed by ST2 (4 datasets; 39.3%, 95% CI 24.9–55.9%), ST1 (8 datasets; 30.9%, 95% CI 19.8–44.7%), ST4 (5 datasets; 13.4%, 95% CI 7.8–22.3%), ST8 (2 datasets; 12.7%, 95% CI 4.6–30.7%), ST10 (5 datasets; 11%, 95% CI 3.8–28%) and ST5 (3 datasets; 8.1%, 95% CI 2.6–22.4%) (Fig. 5). Each of ST6, ST7, ST23 and ST24 was observed in only one study (Table 3). Unlike cats, ST5–8, ST23 and ST24 were only reported in dogs.

**Table 3:** Worldwide distribution of *Blastocystis* subtypes in dogs and cats reported in 19 molecular studies (25 datasets)

Author, year (*n* datasets)	Total samples (*n*)	Infected samples (*n*)	Prevalence (%)	Subtyping of infected samples^a^		Zoonotic subtypes^d^ (*n*/%)
				Subtyped^b^ (*n*/%)	Unidentified^c^ (*n*/%)	
Dogs						
Boutellis, 2021 (2 datasets)	9	1	11.1	–	1/100	–
Osman, 2015	116	4	3.4	ST2 (2/50), ST10 (2/50)	–	Feb-50
Udonsom, 2018 (2 datasets)	13	1	7.7	ST3 (1/100)	–	1/100
Ramirez, 2014	40	15	37.5	ST2 (15/100)	–	15/100
Wang, 2013 (1 dataset)	80	2	2.5	ST1 (2/100)	–	2/100
Wang, 2013 (2 datasets)	80	1	1.3	ST2 (1/100)	–	1/100
Wang, 2013 (3 datasets)	80	19	24	ST1 (9/47.4), ST4 (2/10.5), ST5 (1/5.3), ST6 (7/36.8)	–	19/100
Belleza, 2016	145	20	13.8	ST2 (1/5), ST3 (2/10), ST4 (2/10), ST5 (2/10), ST1/ST3 (1/5), ST2/ST3 (1/5), ST4/ST5 (1/5)	Oct-50	Oct-50
Li, 2016	315	6	1.9	ST1 (2/33.3), ST1/ST2 (4/66.7)	–	6/100
Ruaux, 2014 (2 datasets)	103	10	9.7	ST1 (2/20), ST10 (2/20)	Jun-60	20-Feb
Higuera, 2021	4	2	50	ST23/ST24 (1/50)	Jan-50	–
Gazzonis, 2019	99	21	21.2	ST3 (21/100)	–	21/100
Parkar, 2007 (2 datasets)	20	2	10	ST1 (1/50)	Jan-50	Jan-50
Parkar, 2007 (3 datasets)	3	3	100	ST5 (3/100)	–	3/100
Noradilah, 2017	40	21	52	ST1 (5/23.8), ST3 (7/33.3), ST4 (4/19), ST8 (4/19), ST10 (1/4.8)	–	20/95.2
Liao, 2020	651	35	5.4	ST1 (6/17.1), ST3 (28/80), ST10 (1/2.8)	–	34/97.1
Mohammadpour, 2020 (2 datasets)	154	29	18.8	ST2 (8/27.6), ST3 (11/37.9), ST4 (3/10.3), ST7 (3/10.3), ST8 (2/6.9), ST10 (2/6.9)	–	27/93.1
Wang, 2018	136	4	2.9	ST1 (3/75), ST4 (1/25)	–	4/100
Villamizar, 2019	8	1	12.5	ST1 (1/100)	–	1/100
Cats						
Boutellis, 2021 (1 dataset)	19	12	63.1	ST2 (3/25), ST3 (1/8.3)	8/66.7	4/33.3
Can, 2021	465	17	3.6	ST4 (7/41.2)	10/58.8	7/41.2
Li, 2019	346	2	0.6	ST1 (2/100)	–	2/100
Ruaux, 2014 (1 dataset)	105	12	11.7	ST1 (1/8.3), ST3 (1/8.3), ST10 (4/33.4)	Jun-50	2/16.7
Kwak, 2020	158	1	0.6	ST4 (1/100)	–	1/100
Mohammadpour, 2020 (1 dataset)	119	21	17.7	ST1 (5/23.8), ST3 (7/33.3), ST4 (4/19), ST10 (3/14.3), ST14 (2/9.5)	–	16/76.2

^a^Out of the positive samples of *Blastocystis*,

^b^Some have been subtyped

^c^Some have not been subtyped or not determined

^d^The number and percentage of zoonotic subtypes are computed for ST1-ST8

### Prevalence of each *Blastocystis* subtype in cats

**Fig. 6:**
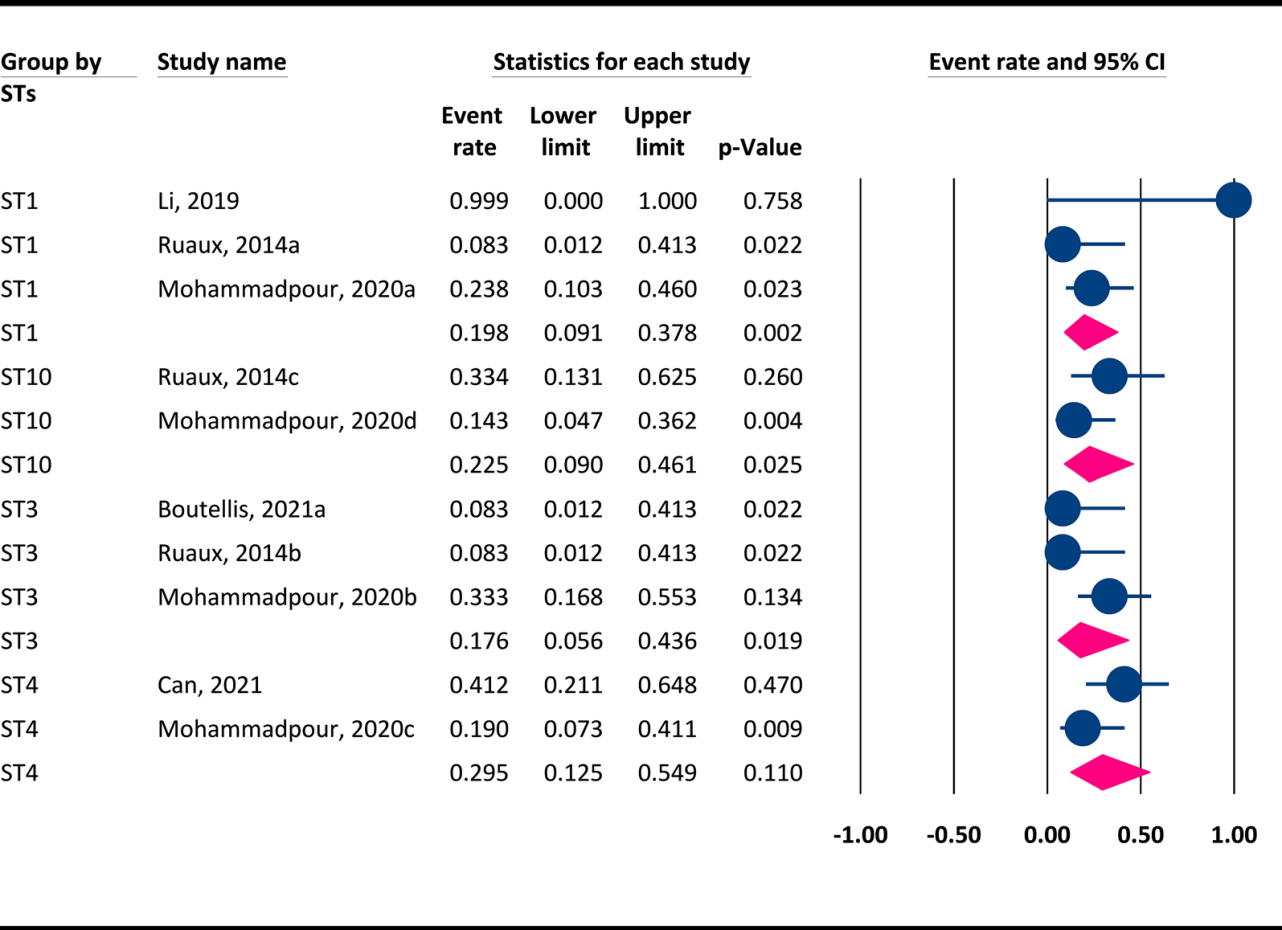
Overall prevalence of each *Blastocystis* subtype in cats using a random-effects model

Relative to dogs, fewer genetically diverse STs were identified in the cats (ST1-4, ST10, ST14). The highest prevalence was observed for ST4 (2 datasets; 29.5%, 95% CI 12.5–54.9%), followed by ST10 (2 datasets; 22.5%, 95% CI 9–46.1%), ST1 (3 datasets; 19.8%, 95% CI 9.1–37.8%) and ST3 (3 datasets; 17.6%, 95% CI 5.6–43.6%) (Fig. 6). Only a single study reported ST2 and ST14, as shown in Table 3. Interestingly, ST14 has only been reported in cats, and there were no reports of dogs being infected with this subtype.

### Uncharacterized *Blastocystis *isolates and zoonotic potential of *Blastocystis* STs in dogs and cats

As shown in Table 3, not all positive samples were characterized in the included studies, possibly leading to underreporting of the true subtype population in both dogs and cats. Of the 10 recognized zoonotic STs of *Blastocystis* (ST1–9, ST12), eight were reported indogs (ST1–8) and four were isolated from cats (ST1–4), suggesting the importance of these animals, particularly dogs, in zoonotic transmission of *Blastocystis* (Table 3).

### Meta-regression

No significant association was found between *Blastocystis* prevalence and sample size in cats (regression coefficient (Reg Coef) = − 0.0033, *P* = 0.101), and publication year in dogs (Reg Coef = − 0.0315, *P* = 0.364). A statistically substantial association was reported between the frequency of *Blastocystis* infection in cats and the year of publication (Reg Coef = − 0.0931, *P* = 0.028), and the sample size in dogs (Reg Coef = − 0.0017, *P* = 0.046) (Additional files 14, 15, 16 and 17: Figures. S13, S14, S15 and S16).

### Publication bias

**Fig. 7:**
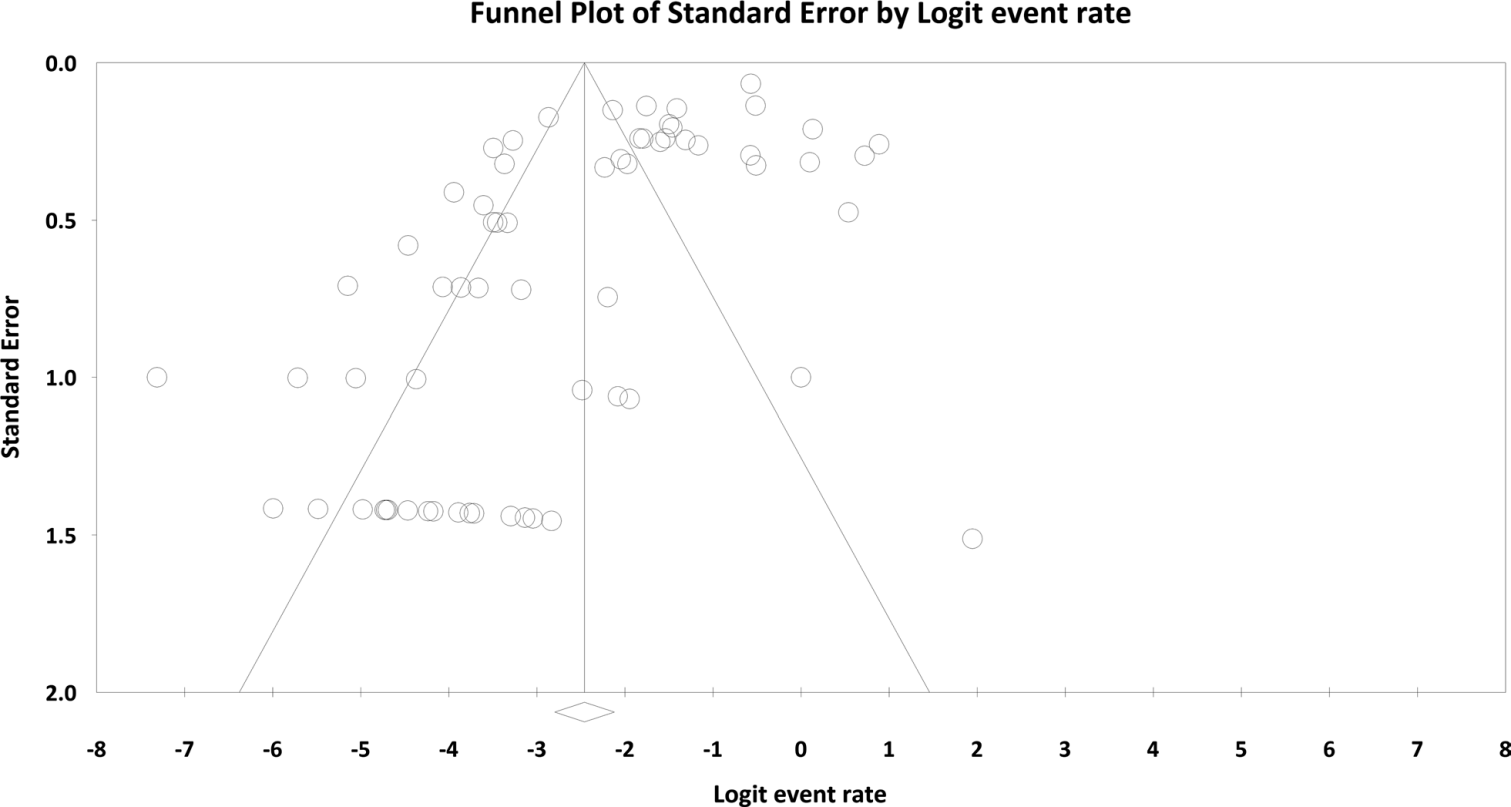
A funnel plot representing publication bias in the present systematic review and meta-analysis

There was a significant publication bias in the present systematic review and meta-analysis (Egger's regression: intercept = − 3.126, 95% lower limit = − 4.412, 95% upper limit = − 1.841,* t*-value = 4.86, *P* < 0.001) (Fig. 7).

## Discussion

The domestication of dogs and cats may be considered as a double-edged sword for humans; these animals are considered to be part of human families on the one hand, but they may carry several zoonotic agents, which can threat human health on the other hand [68]. *Blastocystis* is a zoonotic protozoa that infects a broad range of animals as well as humans [69]. Consequently, prediction of the global prevalence and subtype distribution of *Blastocystis* infection in dogs and cats is of great importance for humans. In the present study, we investigated this subject at a global scale.

Information was extracted from a total of 65 datasets (49 papers) on *Blastocystis* spp. infection in dogs and cats, and pooled frequencies of 9.3% (95% CI 5.3–15.9%) and 7% (95% CI 4.7–10.4%) were estimated for the cat and dog populations included in these datasets, respectively. A true comparison of both groups could not be conducted since a much lower number of studies examined *Blastocystis* spp. infection in cats. Individual studies had no substantial impact on the total prevalence of *Blastocystis* infection, as evidenced by the sensitivity analysis results. An obvious gap exists in terms of comprehensive epidemiological studies on *Blastocystis* infection in animal taxa, and only recently have meta-analyses reported a calculated prevalence rate of 52.4% (95% CI 43.9–60.7%), 31.2% (95% CI 11.2–62%) and 24.4% (95% CI 16.9–33.9%) in domestic pigs, wild boars [9] and cattle [6], respectively. Comparison of findings shows that the global frequency of *Blastocystis* infection is much lower in dogs and cats than in cattle, pigs and wild boars. Possible reasons for such differences may be animal type, number of examined animals, geographical location, among others. Of note, due to the limited number of studies conducted to date, of samples for testing and geographical areas investigated, no accurate comparison can be made and the prevalence rates reported in the present study should be interpreted with caution.

Our findings showed a higher trend of *Blastocystis* prevalence in studies documented up to and including 2000, with a reported prevalence of 23.2% (95% CI 0.3–96.4%)] and 26.1% (95% CI 0.7–94.6%)] for dogs and cats, respectively. Curiously, South America was reported to the most important area for *Blastocystis* in both dogs (6 datasets; 16%, 95% CI 7.7–30.4%) and cats (1 dataset; 37.4%, 95% CI 31.4–43.8%), while the infection was most common among dogs of the AMR region (WHO Region of the Americas; 9 datasets; 14.6%, 95% CI 7.5–26.3%) and among cats of SEAR region (WHO South-East Asian Region; 2 datasets; 23.4%, 95% CI 1.3–87.6%). Although derived from single studies, the highest prevalence was recorded in examined dogs in Chile (36.1%, 95% CI 33.1–39.2%) and India (23.8%, 95% CI 15.7–34.3%), as well as examined cats in Algeria (63.2%, 95% CI 40.3–81.3%). These high prevalences emphasize the importance of this parasitic infection in these countries. Nevertheless, the limited geographical areas studied and the lack of a sufficient number of studies in each country make it impossible for us to make an accurate assessment of the epidemiology of this parasitic infection. Inevitably, the sample size has a large effect on the estimated prevalence of an infection, as reflected in our results: sample sizes of ≤ 50 and 51–100 animals demonstrated the highest prevalence rates for *Blastocystis* infection, a prevalence of 18.6% (95% CI 8.6–35.8%) in dogs and 67.3% (95% CI 5.3.6–78.6%) in cats. It would appear that the results obtained from dogs are more reliable because they have been inferred from several studies (10 datasets), in comparison to the results from cats (1 paper). Taken together, when considering the evaluated subgroups, we found that the confidence intervals of reported frequencies were very wide, which is directly related to the limited number of studies and the large differences in reported prevalence rates. This is obviously a major limitation in our study, which can be eliminated by more comprehensive, nation-wide studies.

Another prominent finding of the present study was that dogs are a crucial source of zoonotic *Blastocystis* subtypes (ST1–ST8) and, therefore, possibly having the potential to transmit such subtypes to humans. However, the number of isolated STs from dogs and cats may increase in the future as not all positive samples in the studies included in this meta-analysis were subtyped. Mixed infections with multiple subtypes are frequently seen in association with *Blastocystis* infection [70]. Mixed cases were reported in some of the studies, but due to various limitations, we could not estimate their pooled prevalence.

Meta-regression results revealed that in contrast to the sample size in cats and publication year in dogs, the year of publication in cats and the sample size in dogs were considered as a cause of variability in *Blastocystis* prevalence. Accordingly, there was a direct association between a reduction in *Blastocystis* infection rate with recently published studies in cats and with an increase in sample size in dogs. A high rate of heterogeneity was reported as publication bias in the present study, which could substantially skew the outcomes [71]. This may originate from differences in geographical region, publication year, number of studies in each area and sample size, as mentioned in Table 2. Other parameters not mentioned in this current review may also represent publication bias, such as the status of animal health, sampling procedures, sample preservation, method of raising owned animals, sensitivity of diagnostic methods, age and sex of the examined hosts and the quality of studies entered. Hence, the results obtained from the present study must be interpreted with caution. In general, despite the valuable epidemiological information we collected in the current study, future studies could, therefore, shed more light on the ST distribution and epidemiological patterns of *Blastocystis* infection in dogs and cats across the globe.

## Conclusion

Currently, many dogs and cats live in the (close) proximity of humans and have the potential to be a threat human health, particularly through zoonotic infections. To the best of our knowledge, we present here the first comprehensive insights into the worldwide epidemiology, subtype distribution and zoonotic potential of *Blastocystis* infection in dogs and cats. The prevalence of this infection was relatively low among dogs (7%) and cats (9.3%), albeit higher higher in cats. Notably, of the 28 reported *Blastocystis* STs, 11 were isolated from dogs and six were isolated from cats, with most of these considered to be zoonotic. Consequently, these animals could play a significant role in the transmission of zoonotic subtypes to humans. The present review was designed and conducted solely on the basis of current published literature (up to 8 November 2021), and more extensive studies are needed to elucidate the epidemiology and distribution of dog and cat STs.

## Supplementary Information

**Additional file 1: Table S1.** JBI critical appraisal checklist applied for included studies

**Additional file 2: Figure S1.** Sensitivity analysis on the pooled* Blastocystis* prevalence in dogs.

**Additional file 3: Figure S2.** Sensitivity analysis on the pooled* Blastocystis* prevalence in cats.

**Additional file 4: Figure S3.** Pooled* Blastocystis* prevalence based on publication year in dogs.

**Additional file 5: Figure S4.** Pooled* Blastocystis* prevalence based on publication year in cats.

**Additional file 6: Figure S5.** Pooled* Blastocystis* prevalence based on continents in dogs.

**Additional file 7: Figure S6.** Pooled* Blastocystis* prevalence based on continents in cats.

**Additional file 8: Figure S7.** Pooled* Blastocysti*s prevalence based on WHO regions in dogs.

**Additional file 9: Figure S8.** Pooled* Blastocystis* prevalence based on WHO regions in cats.

**Additional file 10: Figure S9.** Pooled* Blastocystis *prevalence based on countries in dogs.

**Additional file 11: Figure S10.** Pooled* Blastocystis *prevalence based on countries in cats.

**Additional file 12: Figure S11.** Pooled* Blastocystis* prevalence based on sample size in dogs.

**Additional file 13: Figure S12.** Pooled* Blastocystis* prevalence based on sample size in cats.

**Additional file 14: Figure S13.** Association between* Blastocystis* prevalence and publication year in dogs using meta-regression.

**Additional file 15: Figure S14.** Association between* Blastocystis* prevalence and sample size in dogs using meta-regression.

**Additional file 16: Figure S15.** Association between* Blastocystis *prevalence and publication year in cats using meta-regression.

**Additional file 17: Figure S16.** Association between* Blastocystis* prevalence and sample size in cats using meta-regression.

## Data Availability

The datasets supporting the conclusions of this article are included in the article (and its additional files).
