# Supplementary material for: Current global status, subtype distribution and zoonotic significance of Blastocystis in dogs and cats: a systematic review and meta-analysis
Source: Parasit Vectors. 2022 Jun 22;15:225. doi: 10.1186/s13071-022-05351-2 (PMC9215001; doi:10.1186/s13071-022-05351-2)
Supplement: Supplementary file 1 — Additional file 1: Table S1. JBI critical appraisal checklist applied for included studies [file 13071_2022_5351_MOESM1_ESM.docx]

**Supplementary Table 1**

**JBI critical appraisal checklist applied for included studies**

| Author Name/Year | Sample was representative? | Participants appropriately recruited? | Sample size was adequate? | Study subjects and the setting described? | Data analysis conducted | Objective, standard criteria, reliably used? | Appropriate statistical analysis used | Confounding factors/ subgroups/ differences identified and accounted? | Subpopulations identified using objective criteria | Overall quality |
| --- | --- | --- | --- | --- | --- | --- | --- | --- | --- | --- |
| Abe, 2002 | Yes | Yes | Yes | Yes | Yes | Yes | Yes | No | No | 6/9 |
| Boutellis, 2021b | Yes | Yes | Yes | Yes | Yes | Yes | Yes | No | No | 7/9 |
| Roberts, 2013b | Yes | No | Yes | Yes | Yes | Yes | Yes | No | No | 6/9 |
| Osman, 2015 | Yes | Yes | Yes | Yes | Yes | Yes | Yes | Yes | No | 8/9 |
| Duda, 1998a | Yes | Yes | Yes | Yes | Yes | No | Yes | Yes | No | 7/9 |
| La Sala, 2015 | No | Yes | No | Yes | Yes | No | Yes | No | No | 4/9 |
| Udonsom, 2018b | Yes | Yes | No | No | Yes | No | Yes | Yes | Yes | 6/9 |
| Sardarian, 2015 | Yes | No | Yes | No | No | No | Yes | Yes | No | 4/9 |
| Ramirez, 2014 | Yes | Yes | Yes | Yes | Yes | No | Yes | No | No | 6/9 |
| Sanchez-Thevenet, 2019 | Yes | Yes | No | Yes | Yes | Yes | Yes | Yes | No | 7/9 |
| Wang, 2013a | No | Yes | Yes | Yes | No | No | Yes | No | Yes | 5/9 |
| Puebla, 2015 | No | No | No | Yes | Yes | Yes | Yes | No | No | 4/9 |
| Hurtado, 2019 | No | Yes | No | Yes | Yes | No | Yes | No | Yes | 5/9 |
| Bandaranayaka, 2019 | Yes | Yes | Yes | Yes | Yes | No | Yes | Yes | Yes | 8/9 |
| Spanakos, 2011 | Yes | No | Yes | Yes | No | Yes | Yes | No | Yes | 6/9 |
| Belleza, 2016 | No | Yes | Yes | Yes | Yes | No | Yes | No | No | 5/9 |
| Li, 2016 | Yes | No | No | Yes | Yes | No | Yes | Yes | No | 5/9 |
| Mohaghegh, 2018 | Yes | Yes | Yes | Yes | Yes | Yes | Yes | No | No | 7/9 |
| Ruaux, 2014b | No | Yes | Yes | Yes | No | No | Yes | Yes | Yes | 6/9 |
| Higuera, 2021 | Yes | Yes | No | Yes | Yes | No | Yes | Yes | Yes | 7/9 |
| Gazzonis, 2019 | Yes | No | Yes | Yes | Yes | Yes | Yes | No | No | 6/9 |
| Konig, 1997b | Yes | Yes | No | Yes | Yes | No | Yes | No | No | 5/9 |
| Leelayoova, 2009 | Yes | Yes | Yes | Yes | Yes | No | Yes | Yes | No | 7/9 |
| Dalimiasl, 2001 | Yes | Yes | No | No | Yes | No | Yes | Yes | Yes | 6/9 |
| López, 2006b | Yes | No | Yes | No | No | No | Yes | Yes | No | 4/9 |
| Onder, 2021b | Yes | Yes | Yes | Yes | Yes | No | Yes | No | No | 6/9 |
| Parkar, 2007b | Yes | Yes | No | Yes | Yes | Yes | Yes | No | No | 6/9 |
| Awadallah, 2015 | No | Yes | Yes | Yes | No | No | Yes | Yes | Yes | 6/9 |
| Gonzalez, 2015 | Yes | Yes | No | Yes | Yes | No | Yes | Yes | Yes | 7/9 |
| Gillespie, 2017 | Yes | Yes | Yes | Yes | Yes | No | Yes | Yes | No | 7/9 |
| Hemalatha, 2014b | No | Yes | No | Yes | Yes | No | Yes | Yes | No | 5/9 |
| Noradilah, 2017 | Yes | Yes | No | No | Yes | No | Yes | Yes | Yes | 6/9 |
| Liao, 2020 | Yes | No | Yes | No | No | No | Yes | Yes | No | 4/9 |
| Mohammadpour, 2020b | Yes | Yes | Yes | Yes | Yes | No | Yes | No | No | 6/9 |
| Paulos, 2018b | Yes | Yes | No | Yes | Yes | Yes | Yes | No | No | 6/9 |
| Perera, 2013 | No | Yes | Yes | Yes | No | No | Yes | No | Yes | 5/9 |
| Mokhtar, 2018b | Yes | No | Yes | Yes | Yes | No | Yes | No | No | 5/9 |
| Wang, 2018 | Yes | No | No | Yes | Yes | Yes | Yes | Yes | No | 6/9 |
| Villamizar, 2019 | Yes | Yes | No | Yes | Yes | Yes | Yes | No | No | 6/9 |
| Pagati, 2018 | Yes | Yes | Yes | Yes | Yes | No | Yes | Yes | No | 7/9 |
| Can, 2021 | No | Yes | No | Yes | Yes | No | Yes | Yes | No | 5/9 |
| Badparva, 2020 | Yes | Yes | No | Yes | Yes | Yes | Yes | No | No | 6/9 |
| Arbabi, 2009 | Yes | Yes | Yes | Yes | Yes | Yes | Yes | Yes | No | 8/9 |
| Li, 2019 | No | Yes | Yes | Yes | No | No | Yes | Yes | Yes | 6/9 |
| Khademvatan, 2014 | Yes | Yes | No | Yes | Yes | No | Yes | Yes | Yes | 7/9 |
| Albakri, 2016 | Yes | Yes | Yes | Yes | Yes | No | Yes | Yes | No | 7/9 |
| Okoye, 2014 | Yes | Yes | No | Yes | Yes | Yes | Yes | No | No | 6/9 |
| Kwak, 2020 | Yes | Yes | Yes | Yes | Yes | Yes | Yes | Yes | No | 8/9 |
| Karakavuk, 2021 | No | Yes | Yes | Yes | No | No | Yes | Yes | Yes | 6/9 |
